# MS-Based Characterization of Biomass-Derived Materials: Activated Carbons and Solvent Liquefaction Products

**DOI:** 10.3390/polym17030258

**Published:** 2025-01-21

**Authors:** Cristian D. Gutierrez Reyes, Sherifdeen Onigbinde, Ananda S. Amarasekara, Moyinoluwa Adeniyi, Joy Solomon, Herson A. Gonzalez Ponce, Yehia Mechref

**Affiliations:** 1 Department of Chemistry and Biochemistry, Texas Tech University, Lubbock, TX 79409, USA; cristian.d.gutierrez-reyes@ttu.edu (C.D.G.R.); sonigbin@ttu.edu (S.O.); moadeniy@ttu.edu (M.A.); joy.solomon@ttu.edu (J.S.); 2 Department of Chemistry, Prairie View A & M University, Prairie View, TX 77446, USA; asamarasekara@pvamu.edu; 3 Division of Graduate Studies and Research, Instituto Tecnológico de Aguascalientes, Tecnológico Nacional de México, Aguascalientes 20255, Mexico; herson_qfbd@hotmail.com

**Keywords:** mass spectrometry, biomass, solvent liquefaction, activated carbons

## Abstract

Mass spectrometry (MS) is a powerful analytical technique that is widely used to characterize a variety of analytes across diverse fields. In the area of biomass conversion, which is essential for producing sustainable materials and energy, the role of MS is pivotal. Biomass conversion processes, such as solvent liquefaction and pyrolysis, generate a wide range of industrially valuable materials including bio-based polymers, fuels, and activated carbons. However, the inherent complexity and heterogeneity of biomass and its transformation products pose significant analytical challenges. Advanced MS techniques, such as GC-MS, LC-MS, ICP-MS and MALDI-MS, are essential for a comprehensive analysis, providing detailed insights into the compositions, impurities, and potential inhibitors that influence process optimization and product quality. This review systematically explores recent advancements in MS-based methods for the analysis of biomass-derived products. We discuss fundamental innovations in biomass conversion processes and highlight the applications of various MS techniques in assessing the chemical complexity of these materials. The insights provided by MS techniques not only facilitate process optimization but also support the sustainable production of high-value materials from biomass, aligning with ongoing efforts to enhance environmental sustainability and resource efficiency.

## 1. Introduction

Biomass is a complex and heterogeneous material composed of various organic compounds, such as cellulose, hemicellulose, and lignin [1,2,3]. Its elemental composition, which typically includes carbon, hydrogen, oxygen, nitrogen, and sulfur, varies significantly depending on the source, such as agricultural residues, forestry by-products, or algae [4,5]. This variability influences the efficiency and outcomes of biomass conversion processes, including pyrolysis and solvent liquefaction [6,7].

Mass spectrometry, in particular, stands out as a versatile and highly sensitive tool for analyzing the complex mixtures generated during biomass conversion [8,9]. Advanced MS techniques, including GC-MS, liquid chromatography–mass spectrometry (LC-MS), ICP-MS, and matrix-assisted laser desorption/ionization (MALDI-MS), enable the detailed profiling of biomass-derived products [10,11,12,13,14,15,16,17,18,19]. GC-MS is widely used for analyzing the volatile and semi-volatile compounds produced during biomass pyrolysis and gasification [20,21]. This technique can identify a wide range of organic molecules, including hydrocarbons, aldehydes, ketones, phenols, and furans. In activated carbon analysis, GC-MS helps to assess the adsorbed organic compounds and residual tars, providing insight into the material’s effectiveness for adsorption applications [22,23,24]. Analytical tools, such as Fourier transform infrared (FTIR) spectroscopy and thermogravimetric analysis (TGA), are commonly utilized in combination with GC-MS for studying the formation of gasses, volatiles, and intermediates [25,26,27,28,29,30,31,32,33]. These analyses allow researchers to fine-tune reaction conditions to maximize yields and minimize undesirable by-products. LC-MS is ideal for analyzing non-volatile and thermally labile components, such as bio-oils and liquefaction products [30]. It is beneficial for identifying complex mixtures of high-molecular-weight compounds, such as lignin-derived oligomers, organic acids, and bioactive compounds in solvent liquefaction products [34,35]. In helping to understand the molecular composition and reaction dynamics, these techniques are instrumental in advancing biomass’s sustainable and efficient transformation into valuable industrial products [28].

The presence of impurities like ash, heavy metals, and salts further complicates these processes, often reducing catalyst performance and affecting the quality of the end products [36,37]. ICP-MS is essential for detecting and quantifying trace elements in biomass and its derivatives, such as heavy metals and inorganic impurities [38,39,40,41,42]. These impurities can influence the catalytic efficiency of biomass conversion processes and the quality of the final products. ICP-MS allows for the precise determination of elemental compositions, enabling researchers to address contamination issues and improve process outcomes. MALDI-MS is particularly useful for analyzing large biomolecules, such as lignin and its derivatives, in biomass-derived products [43,44,45]. This technique allows us to ionize high-molecular-weight species without fragmentation, making it ideal for studying the molecular structure of lignocellulosic materials and the products of solvent liquefaction.

**Figure 1:**
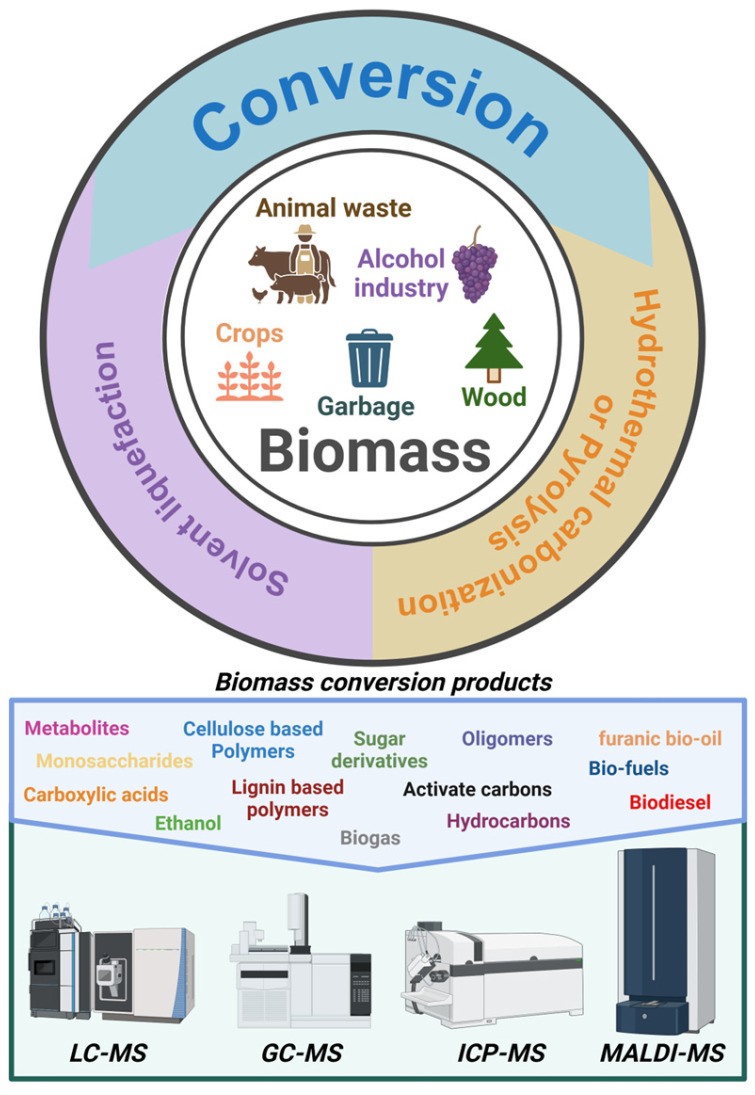
Representative workflow. Different types of biomasses are converted into a variety of products that are analyzed according to their physicochemical properties.

The MS-based characterization of biomass-derived materials, such as activated carbons and solvent liquefaction products, is essential for understanding their chemical composition and identifying impurities and inhibitors in both the raw biomass and its conversion products. Advanced MS techniques provide researchers with critical insights into the complex mixtures generated during biomass conversion processes. Real-time monitoring, enabled by these methods, allows for the optimization of processes such as pyrolysis and gasification, ultimately enhancing the efficiency and sustainability of biomass-derived material production, see Figure 1.

As the demand for renewable and sustainable resources continues to grow, the role of MS-based analytical techniques in advancing biomass conversion technologies is becoming increasingly significant. This review emphasizes the importance of mass spectrometry (MS) in characterizing biomass-derived materials and focuses on how these techniques are employed to analyze the intricate mixtures formed during conversion processes like pyrolysis, gasification, and solvent liquefaction. Specifically, it explores the utility of GC-MS, LC-MS, ICP-MS, and MALDI-MS in providing a deeper understanding of the chemical composition, impurities, and inhibitors associated with biomass conversion. Such insights are crucial for optimizing conversion processes and advancing the development of efficient, sustainable methods of producing valuable materials from biomass.

## 2. Biomass Derived Activated Carbons

Biomass-derived activated carbon (BDAC) is highly valued for its exceptional surface area, porosity, and chemical stability, making it widely applicable across various fields. BDAC is extensively utilized as an adsorbent for removing environmental pollutants from water [46], air [47], and soil [48]. It also serves as a key material in energy storage applications, such as capacitors and supercapacitors [49,50], and demonstrates biological activity through its antibacterial, antioxidant [51], and pharmacological properties [25,52]. Additionally, BDAC is a catalyst support in electrochemical processes and biofuel production [53,54].

Numerous studies have demonstrated its versatility. Guo and coworkers [55] utilized a corncob-derived activated carbon prepared through KOH activation–carbonization, achieving a high specific surface area of 2308 m^2^/g and an adsorption capacity of 523 mg/g for methylene blue dye, with a removal rate of 99%. This material showed excellent durability, maintaining its performance over repeated use. Similarly, Cheng et al. [56] developed cotton stem-derived activated carbon (CSA) using microwave-assisted activation with K_2_CO_3_. With a specific surface area of 2017 m^2^/g and mesoporous structure, CSA exhibited a maximum adsorption capacity of 265 mg/g for Rhodamine B dye and retained high efficiency over multiple cycles. These processes’ byproduct gasses (primarily H_2_ and CO) were also captured for potential use as fuels, enhancing sustainability.

Other lignocellulosic agricultural wastes, such as sugarcane bagasse and sawdust, produce activated carbon for use in pollutant removal. Thabet and coworkers [57] employed a combination of physical and chemical activation methods (water vapor, K_2_CO_3_, and KOH) to create activated carbon with a surface area of up to 2490 m^2^/g and a microporous structure. This material effectively removed phenol and methylene blue, providing a cost-effective solution for water purification. Activated carbon from coconut shells [58], rice straw [59], orange peel [60], and cashew nut shells [61] has also been successfully applied in wastewater treatment.

BDAC has gained increasing importance in energy storage, particularly in supercapacitors and batteries [62,63]. Its high surface area and conductivity make it ideal as an electrode material, improving the energy storage capacity of devices [64]. Supercapacitors from BDAC offer a sustainable and cost-effective alternative to traditional materials and demonstrate significant performance improvements [65,66,67,68,69,70]. For instance, Hegde and Bhat [71] produced activated carbon from mango leaf waste. This was optimized at 725 °C, achieving a specific surface area of 1232 m^2^/g and a capacitance of 521 F/g. The material retained 97% capacitance after 10,001 cycles, highlighting its stability for high-performance energy storage devices. Similarly, Numee et al. [72] used durian shell biomass, treated with gamma and electron beam irradiation, to produce activated carbon with a specific capacitance of 325.20 F/g and 94.79% retention after 10,000 cycles. Okoye et al. [73] created activated carbon from corn husk biomass, yielding a specific surface area of 1115 m^2^/g and capacitances up to 269 F/g, maintaining 99% capacity over 20,000 cycles.

Beyond energy storage, BDAC emerged as a sustainable catalyst in various chemical reactions due to its porous structure, high surface area, and functional groups that enhance catalytic activity [74]. Tailored BDAC catalysts, incorporating acidic, basic, or metal-impregnated active sites, are employed in oxidation, reduction, and dehydration reactions [75]. For example, BDAC can support metal catalysts during hydrogenation or oxidation processes in environmental remediation, biofuel production, and fine chemical synthesis [76]. Its eco-friendliness, low cost, and high tunability make BDAC a promising alternative to traditional catalysts in green chemistry and industrial applications [77]. For example, Zhao et al. [78] utilized lipid-extracted microalgae biomass (LMB) catalyzed by waste eggshell-derived CaO to enhance hydrogen production while reducing CO_2_ emissions. Nabi and coworkers [79] developed a rice husk-derived solid acid catalyst (RH-SO_3_H) that efficiently hydrolyzed corncob lignocellulose into reducing sugars, yielding 486 mg/g under optimized conditions. A study by Li et al. [80] used corncob-derived activated carbon loaded with nickel monoxide (NiO) to convert waste cooking oil into bio-oil with a 99% hydrocarbon content, demonstrating an eco-friendly approach to fuel production.

Comprehensive reviews, such as that by Quevedo-Amador et al. [81], summarize the diverse applications of biomass-derived catalysts. These studies underscore BDAC’s versatility, sustainability, and economic potential in addressing environmental and industrial challenges.

## 3. Solvent Liquefaction of Biomass

The growing interest in sustainable transportation fuels has fueled significant research into the conversion of lignocellulosic biomass into biocrude oil, which can be further refined into renewable hydrocarbon fuels [82]. Various techniques are currently under investigation, including pyrolysis [83], hydrothermal liquefaction [84,85,86], organic solvent liquefaction [87,88], the catalytic processing of algae oils [89,90,91], bio-ethylene oligomerization [92], and syngas oligomerization [93,94]. These liquefaction processes can be conducted in different atmospheres, such as air, nitrogen, or hydrogen. Notably, liquefaction under a hydrogen atmosphere is a promising advancement, as catalytic reductive deoxygenation facilitates the in situ conversion of oxygenated intermediates into alkanes.

The bio-oils or biocrude oils produced through these methods are complex mixtures of compounds, including alkanes, alkenes, alcohols, aldehydes, carboxylic acids, phenols, and other aromatics. The composition of these oils depends heavily on the conversion technique employed. For example, the hydrothermal and organic solvent liquefaction of lignocellulosic biomass primarily yields aliphatic products derived from cellulose and hemicellulose polysaccharides, alongside aromatic compounds such as phenols and aromatic alcohols from lignin. Conversely, bio-crude derived from cellulosic ethanol [95] involves alumina-catalyzed dehydration into ethanol and ethylene, followed by oligomerization into alkanes and alkenes. Similarly, the syngas oligomerization approach results in alkanes, alkenes, and alcohol mixtures.

In recent years, research on biomass liquefaction has increasingly focused on producing sustainable aviation fuels, or bio-jet fuels, as detailed in several comprehensive review articles [96,97,98]. In addition to the direct liquefaction of cellulosic biomass into bio-crude, a burgeoning area of interest involves the carbon number upgrading of cellulose-derived feedstocks such as furfural, 5-hydroxymethyl furfural (HMF), and levulinic acid [28,99,100].

Since bio-crude is a complex mixture of compounds, typically ranging from C4 to C15, with diverse functional groups, its complete characterization poses significant challenges. Nonetheless, separation techniques coupled with mass spectrometry, such as GC-MS and LC-MS, are widely employed to identify and quantify its components. Additionally, ^1^H NMR spectroscopy is used to estimate the composition of compound classes within bio-oils. For instance, ^1^H NMR integration is often applied to determine the ratio of aromatics, alcohols/ethers, and alkenes in bio-crude oils [101,102]. Beyond chemical characterization, physical properties like density, viscosity, flash point, and higher heating values are critical industry standards for evaluating biomass-derived oils [103].

GC-MS is particularly suited for analyzing more volatile, low-molecular-weight alkanes, alkenes, and alcohol mixtures formed during syngas or bio-ethylene oligomerization processes. On the other hand, liquid chromatography, operating at relatively lower temperatures, is ideal for separating the more complex mixtures produced during hydrothermal and organic solvent liquefaction. These methods handle the diverse functionalities of oxygenated compounds resulting from the complexity of lignocellulosic biomass feedstocks.

## 4. MS-Based Techniques Used for the Characterization of Biomass Derived Products

Biomass conversion processes produce complex mixtures, necessitating the development of precise and reliable mass spectrometric (MS) techniques for their structural and compositional characterization. Among the available MS technologies, quadrupole analyzers are widely used due to their simplicity, high scanning speeds, affordability, and compatibility with gas chromatography (GC) and liquid chromatography (LC) systems. Their low vacuum requirements further contribute to their widespread application. However, quadrupoles have limited mass resolutions, making them more suitable for routine analyses rather than the characterization of highly complex samples [104].

Ion traps (ITs), including linear ion traps, provide higher resolutions than quadrupoles and allow tandem mass spectrometric (MS^n^) experiments. This capability is particularly valuable for identifying unknown compounds in products derived from biomass processes like solvent liquefaction [28]. Time-of-flight (TOF) analyzers, known for their high-speed scanning and broad mass range, offer enhanced resolutions compared to quadrupoles. These attributes make TOF analyzers well suited for detecting the diverse array of molecules present in biomass-derived products [105].

Orbitrap and Fourier-transform ion cyclotron resonance (FT-ICR) mass analyzers provide unparalleled mass accuracy and resolution for advanced structural elucidation. These instruments are essential for analyzing complex molecular compositions, enabling detailed investigations of biomass conversion products. The selection of a mass analyzer ultimately depends on the analytical requirements of the biomass-derived sample, striking a balance between sensitivity, resolution, and economic considerations to ensure reliable and efficient characterization.

Tandem mass spectrometry (MS/MS) further enhances the capabilities of traditional MS by incorporating multiple mass analyzers, significantly increasing specificity and sensitivity. In MS/MS, an ion of interest is selected in the first mass analyzer and then fragmented by collision with inert gas atoms in a collision cell. The resulting fragment ions are separated in a subsequent mass analyzer, enabling detailed structural analysis and characterization. This approach is especially effective for complex molecular investigations, making it a cornerstone of modern analytical workflows for biomass-derived materials.

### 4.1. Liquid Chromatography–Mass Spectrometry (LC-MS)

LC-MS provides a significant advantage over GC-MS due to its applicability to a broader range of compounds, from volatile to non-volatile, polar, and structurally complex biomolecules, making it a versatile tool for diverse applications. It is particularly critical in studying solvent liquefaction products, which often consist of thermally transformed, large biomass-derived molecules [15,106]. The main components of an LC system include several critical parts that work together for efficient sample analysis. The Mobile Phase Reservoir contains liquids that carry the analytes through the system. The choice of solvent and stationary phase depends on the specific type of analysis being performed. The LC pump regulates the flow rate and pressure of the mobile phase, ensuring that samples are carried steadily through the system. The injector introduces the sample into the mobile phase stream, allowing it to enter the LC system. The column contains the stationary phase, where analyte separation occurs based on interactions with the column material. The MS serves as a final detector that identifies and quantifies the analytes, completing the analysis by providing data on each component in the sample. In the MS, the sample/analyte goes through a series of essential components constituting the ionization source, where samples are converted into charged entities/ions. Charged ions are analyzed in mass analyzers and finally detected by detectors. LC-MS/MS enhances the separation and detection of analytes by separating them from matrix components and interfering compounds, leading to improved sensitivity, precision, and specificity [107].

By enabling precise molecular identification, LC-MS enhances our understanding of these complex mixtures, thereby facilitating the optimization of biomass liquefaction processes and the functionalization of activated carbons for environmental and industrial applications.

LC-MS/MS is employed to detect soluble products formed during the hydrothermal conversion of furanic compounds such as 5-hydroxymethylfurfural (HMF), furfural, and furfuryl alcohol [15]. This technique is also instrumental in characterizing the chemical transformations involved, shedding light on the formation of polymeric structures and intermediates during biomass processing [15]. For example, Shi et al. used LC-MS/MS to investigate water-soluble products from the hydrothermal carbonization (HTC) of glucose, identifying compounds such as 3-deoxyglucosone, HMF, levulinic acid, and newly discovered carbocyclic oxy-organics with hydroxy and carbonyl groups [106]. Additionally, LC-MS is utilized to analyze dilute acid-pretreated biomass hydrolysates, quantifying 24 organic acids with minimal sample volume and straightforward preparation protocols, ensuring efficient and accurate results [108].

Amarasekara and coworkers explored cellulose degradation in acetone using acidic ionic liquids as catalysts [28]. The reaction products, analyzed with RP-LC-MS, revealed eleven compounds resulting from cellulose degradation and the aldol condensation of HMF with one to three acetone molecules. Biomass samples from corncobs and switchgrass were subsequently tested, achieving conversion rates of 63.4 ± 0.9% and 56.4 ± 0.9% (*w*/*w*), respectively. Polar fractions were analyzed using LC-MS, while non-polar fractions were characterized by GC-MS, demonstrating the formation of cross-aldol condensation products derived from furfural, HMF, and acetone [30].

LC-MS is also employed to characterize biocrude produced from biomass liquefaction catalyzed by acidic ionic liquids, identifying eleven key compounds, including HMF, its aldol condensation products with acetone, and furfural derivatives. By analyzing total ion chromatograms (TICs) and comparing responses to HMF standards, researchers quantified these compounds, providing insights into the catalytic efficiency of ionic liquids in biomass degradation and the pathways for upgrading biocrude [28].

In studies on the effects of ethanol–water mixtures on biomass liquefaction products, as well as LC-MS and GC-MS analyses, revealed various degradation products, such as levulinic acid, acetic acid, formic acid, and ethyl levulinate. These findings highlighted the synergistic effect of ethanol and water in enhancing liquefaction efficiency, optimizing the breakdown of biomass into valuable chemicals [109]. Fernandes et al. employed LC-MS to analyze bio-oil from the liquefaction of eucalyptus globulus sawdust and bark, identifying high-value compounds such as levulinic acid and furfural, alongside cellulose- and hemicellulose-derived degradation products, providing detailed insights into the molecular transformations during the process [110].

A study by Saengsen et al. [111] optimized the detection and purification of levulinic acid (LA) and related bio-compounds from oil palm empty fruit bunch (OPEFB) biomass using LC-MS/MS and HPLC-DAD. LC-MS/MS demonstrated superior sensitivity for non-chromophoric substrates, while HPLC-DAD effectively identified chromophoric compounds, resulting in a scalable approach for LA purification and the sensitive analysis of intermediates like HMF and furfural. Another study evaluated the biochar produced from sulfuric acid-pretreated biomass for adsorbing diazinon, a hazardous organophosphate pesticide. LC-MS/MS ensured the precise quantification of diazinon concentrations, demonstrating the biochar’s efficiency in removing pesticides from contaminated soil [112].

Lin and coworkers utilized LC-MS/MS to characterize the green synthesis of 5-HMF from biomass-derived carbohydrates, specifically D-glucose, using deep eutectic solvents (DESs) as catalysts under microwave-assisted heating. This method enabled the accurate quantification of 5-HMF yields [113]. Recently, Kabir et al. used LC-MS/MS to evaluate reduced graphene oxide (GrGO) synthesized from waste dry-cell batteries using jute leaf extract as a reducing agent. GrGO showed exceptional adsorption performance, removing up to 98% of antibiotics such as tetracycline, oxytetracycline, and chlortetracycline from aqueous solutions, with LC-MS/MS enabling precise concentration measurements before and after adsorption [114].

### 4.2. Gas Chromatography–Mass Spectrometry (GC-MS)

Biomass and its derived products are inherently complex and heterogeneous, making their analysis and characterization particularly challenging [115]. Pyrolysis, a thermochemical process, converts biomass into gasses, liquids, and char in the absence or near absence of oxygen [115,116]. This process is pivotal for understanding the dynamics of biomass reactions and characterizing the resulting products through methods like evolved gas analysis and thermogravimetric analysis. Gas chromatography–mass spectrometry (GC-MS) plays a crucial role in this context, providing the precise separation, detection, and quantification of both volatile and low-molecular-weight non-volatile compounds [117,118]. In the GC section, a sample is introduced through a heated injector, where it vaporizes and mixes with an inert carrier gas (e.g., helium or nitrogen). The carrier gas transports the sample into the column, where separation occurs as analytes interact with the stationary phase based on their volatility and chemical affinity. The column oven precisely controls temperature, either isothermally or through a temperature gradient, to optimize separation for complex mixtures. Separated analytes are then carried through a heated transfer line into the detector, MS [118,119].

Mass spectrometry enables the identification of compounds and the deduction of molecular structures through the ionization and fragmentation of molecules, making it particularly effective for analyzing volatile biomass-derived materials [104,117].

Fast pyrolysis efficiently converts dry biomass into liquid biofuel, achieving yields of up to 70% by weight, employing additional by-products such as carbon-rich char [116,120,121]. While the low surface area of char limits direct applications, chemical activation can transform it into a valuable porous carbon material. These activated carbons find applications as bioadsorbents, catalyst supports, and electrode materials, extending their utility beyond energy generation [115,122,123]. Pyrolysis, combined with GC-MS, has been extensively utilized to study structural degradation behavior, quantify bio-crude yields, and characterize solid and gaseous products. For instance, Hwang et al. investigated the structural degradation of fast pyrolysis char [116], while Castellvi Barnes et al. characterized and quantified bio-crude, solids, and gasses produced during pyrolysis [124]. GC-MS analysis also revealed the distinct chemical compositions of biomass tar compared to fossil-derived tars and pitches [125].

In specific applications, GC-MS/MS is used to analyze harmful by-products such as benzo[α]pyrene tetrol, a metabolite of the polycyclic aromatic hydrocarbon benzo[α]pyrene that is formed during the incomplete combustion of organic matter like biomass [126]. This technique allowed Pilz et al. [126] to quantify benzo[α]pyrene metabolites in urine, offering insights into human exposure to harmful pyrolysis by-products.

Amarasekara and coworkers demonstrated a one-pot system for biomass depolymerization, product condensation, and cross-aldol reactions using ionic liquid catalysts. The resulting bio-oil, analyzed via GC-MS, revealed 64 products ranging from 4 to 17 carbon atoms, including partially reduced and deoxygenated furans [28,30]. The products from this reaction are highly oxygenated molecules, which are consequently subjected to a further hydrogenation protocol using H_2_, 1 Atm, 23 °C, and 5% Pd-C as catalysts [29]. Similarly, acidic ionic liquids used in sawdust liquefaction produced bio-oils with lighter fractions rich in octyl ethers, esters, and derivatives, as revealed by GC-MS analysis. These results indicated the predominance of cellulose- and hemicellulose-derived compounds with minimal lignin contributions, forming fuel-like, high-octane products [127].

GC-MS is also integral in characterizing bio-oils from acidic ionic liquid-catalyzed cellulose liquefaction. Three major products were identified, including 2-hydroxyethyl levulinate and a novel cellulose-derived compound, 2, 3, 6, 7-tetrahydro-cyclopenta [1,4] dioxin-5-one. The consistent composition of these products over extended reaction times suggests a steady-state equilibrium [128]. Similarly, furanic bio-crude oils from corn stover biomass were analyzed via GC-MS, identifying complex aldol condensation products derived from acidic ionic liquid-catalyzed biomass liquefaction [95].

GC-MS is also employed to analyze light oil from woody biomass liquefaction in polyhydric alcohol environments. This analysis revealed compounds such as glycol derivatives, esters, aromatic compounds, and cellulose degradation products, highlighting the technique’s role in identifying intermediate and final liquefaction products [129]. Furthermore, the composition of bio-oils derived from sewage sludge varied significantly with solvent choice. Ethanol-based liquefaction favored esters, while water promoted organic acid formation, demonstrating how solvent selection influences the chemical profile and suitability of bio-oils as fuel sources [130].

In a comparative analysis of biomass liquefaction feedstocks, GC-MS results showed distinct compound profiles. Rice straw yielded predominantly phenolic compounds, while sewage sludge and *Spirulina* biomass produced mainly esters. This variation underscores the importance of feedstock selection in optimizing bio-oil composition and yield [131]. Ramsurn and Gupta employed GC-MS to characterize energy-dense bio-crudes obtained from switchgrass liquefaction using a two-step process involving, acidic subcritical and alkaline supercritical water, demonstrating the approach’s effectiveness in enhancing bio-crude quality [132].

Innovative liquefaction methods, such as Raney nickel and sodium hydroxide-catalyzed one-step processes, are used to produce phenols, acids, alcohols, hydrocarbons, and ketones. GC-MS enabled the precise characterization of these products, revealing their molecular weights and structural features [133]. For lignin depolymerization, GC-MS identified and quantified a diverse range of compounds, elucidating reaction pathways and highlighting key parameters like alkylation and deoxygenation extent, which are essential for transforming lignin-derived phenolics into high-value chemicals and fuels [134].

### 4.3. Inductively Coupled Plasma—Mass Spectrometry (ICP-MS)

Mass spectrometry is one of the most widely used analytical tools, with recent advancements in ionization techniques, mass analyzers, and detectors further expanding its applications. Among these techniques, inductively coupled plasma mass spectrometry (ICP-MS) stands out for its suitability in analyzing biomasses and biomass-derived products [135]. A diluted or thermally digested sample is often introduced into a nebulizer as a liquid in the form of a true solution. It is then converted into an aerosol and carried into the ICP using an inert carrier gas such as argon. Within the plasma, the sample aerosol is subjected to extremely high temperatures, ranging from approximately 6000 to 10,000 K. This atomizes and ionizes the sample elements into positively charged ions. These ions are then extracted into the mass spectrometer through cones that focus and reduce the ion beam [136].

ICP-MS enables the rapid and precise quantification of trace and major elements by ionizing sample atoms in a plasma and detecting them based on their mass-to-charge ratio [136]. This capability is particularly valuable for understanding the mineral content and potential contaminants in biomass used for bioenergy production [13,137]. Its ability to measure a broad range of metals and metalloids, even at low concentrations, makes it an indispensable tool in biomass research. Additionally, ICP-MS provides insights into the release patterns of specific elements under various conditions, such as combustion and gasification, which are crucial for addressing ash-related challenges and minimizing environmental impacts [39].

The high sensitivity, speed, and broad detection range of ICP-MS make it ideal for biomass analysis. For example, Kachroo et al. [14] employed ICP-MS to investigate the composition and spatial distribution of organic and metallic elements in rice husk, rice straw, and bamboo chips to optimize thermochemical conversion processes. Their study revealed significant variations in inorganic content, with rice residues containing high silicon levels, which could lead to slagging and equipment fouling. In contrast, bamboo’s higher potassium concentration and uniform composition suggested more efficient thermal conversion and reduced ash formation. These findings underscore the importance of tailoring biomass processing to enhance bioenergy production efficiency while mitigating operational issues.

Kienzl and coworkers [39] utilized ICP-MS coupled with a single-particle reactor (SPR) to analyze the release behavior of potassium (K) and sodium (Na) during biomass combustion at high temperatures (1000 °C). This approach allowed the precise monitoring of alkali metal release during the devolatilization stage, showing that higher alkali concentrations in biomass samples corresponded to increased emission intensities and gas phase release rates. Their findings, corroborated by flame emission spectroscopy (FES), are critical for optimizing combustion processes and minimizing emissions.

In another study, Singh et. al. [138] used ICP-MS to measure the elemental composition of torrefied *Acacia nilotica* biomass, focusing on elements like sodium, potassium, calcium, and magnesium. The analysis demonstrated that torrefaction enriched these elements, with concentrations increasing at higher torrefaction temperatures. These compositional data suggest that torrefied biomass has potential in soil amendment, due to its enhanced mineral content, and as a fuel with improved combustion properties.

ICP-MS is often combined with other analytical methods to provide comprehensive insights into biomass processes. Paulauskas et al. [39] integrated ICP-MS with thermogravimetric analysis (TGA) to study the release of heavy metals during the gasification of plant-assisted bioremediation (PABR) biomasses [139,140,141]. TGA simulated gasification conditions, while ICP-MS measured the release of metals such as Fe, Ni, and Pb into the gas phase at specific temperature stages. This approach provided detailed profiles of metal emissions based on the type of bed material used, such as olivine, K-feldspar, kaolinite, or calcite. This study highlighted the role of bed materials in controlling metal emissions and improving syngas quality. Additional applications of ICP-MS include the analysis of the elemental composition of microalgae-based biochar obtained through pyrolysis, as discussed in the works of other researchers [139,140,141]. These studies demonstrate ICP-MS’s versatility and importance in advancing biomass research, from understanding elemental dynamics to optimizing conversion processes and evaluating by-products for environmental and industrial applications.

### 4.4. Matrix-Assisted Laser Desorption/Ionization—Mass Spectrometry (MALDI-MS)

MALDI-MS is particularly well suited for analyzing high-molecular-weight biomolecules, such as peptides and polysaccharides found in biomass. In the MALDI-MS system, the sample is mixed with a chemical matrix, which absorbs laser energy and facilitates ionization. The sample–matrix mixture is applied onto a target plate and allowed to crystallize. The target plate is then placed in the ionization source, where a pulsed laser beam excites the matrix, causing the desorption and ionization of the sample molecules into the gas phase. The generated ions are directed into the mass analyzer and the detector [142]. This technique enables low sample consumption, supports high-throughput screening and facilitates direct MS imaging applications [142]. By accommodating a broad spectrum of chemical polarities and molecular sizes, MALDI-MS significantly enhances the analytical coverage of biomass-derived products, making it a powerful tool for their characterization.

Mase et al. [44] demonstrated the superiority of MALDI-MS over laser desorption ionization for the analysis of lignocellulosic biomass-based bio-oils. The study evaluated twelve matrices, including proton-transfer and electron-transfer types, and identified dithranol, acetosyringone, and graphene oxide as optimal for detailed molecular characterization. These matrices enabled ionization across a wide range of polarities, aromaticities, and molecular masses with consistent relative intensities, streamlining the selection of suitable matrices for specific analyses.

Environmental weathering and chemical degradation introduce impurities and increase the chemical complexity of lignin, posing challenges for its characterization and utilization. Qi et al. [11] used MALDI-MS to investigate the chemical diversity of lignin degradation products, employing matrices such as DHB, CHCA, and DCTB, which exhibited distinct selectivity for sulfur- and nitrogen-containing lignin species. This study provided valuable insights into lignin’s structural heterogeneity.

In another application, MALDI-MS was used to evaluate the distribution of 4-O-methylglucuronic acid residues in xylans, revealing distinct differences in structural patterns between softwoods and hardwoods. Softwood xylans exhibited a periodic distribution of these residues along the polysaccharide chain, whereas hardwood xylans displayed an irregular arrangement. The high sensitivity of MALDI-MS, coupled with its ability to minimize sample fragmentation, enabled precise mass determination and yielded detailed structural insights [143].

Brasseur and colleagues [141] utilized MALDI-TOF MS to identify and structurally profile oligosaccharides such as cellodextrins and xylo-oligosaccharides derived from cellulose and xylan, respectively. This approach enabled the precise determination of molecular weights and degrees of polymerization, offering insights into composition and structural modifications. The study highlighted MALDI-MS as a valuable tool for investigating enzymatic hydrolysis mechanisms in biomass materials.

Reyes-Weiss et al. [12] employed MALDI-MS to analyze fucoidan hydrolysates and screen endo-fucoidanase activity, focusing on complex sulfated polysaccharides derived from brown macroalgae. Using endofucoidanases P5AFcnA and Wv323, the researchers enzymatically degraded fucoidans into defined oligosaccharides. MALDI-MS offered a high-throughput, sensitive method for detecting these oligosaccharides directly from untreated biomass or commercial fucoidan extracts. The study revealed unique oligosaccharide fingerprints, reflective of structural variations across species and environmental conditions, enhancing our understanding of fucoidan structure–function relationships and enzymatic transformations.

Barbano et al. [143] demonstrated the utility of MALDI-TOF MS in characterizing microalgae and their mixtures. This technique effectively differentiated microalgae at the species and strain levels, surpassing traditional methods like 18S rDNA sequencing. It also identified mixture-specific peaks that could serve as biomarkers of contamination or interspecies interactions. MALDI-TOF MS’s sensitivity and efficiency make it a promising tool for monitoring the health and productivity of large-scale microalgae production systems.

In a comprehensive review, Gopal and Muthu [144] highlighted the application of MALDI-TOF MS in analyzing mushroom polysaccharides, which hold significant clinical, nutritional, and medicinal value. They emphasized MALDI-TOF MS’s advantages, including its ability to rapidly and precisely detect polysaccharide molecular structures and macromolecules. The technique was particularly effective in identifying biologically active compounds such as β-glucans and heteropolysaccharides from various mushroom species. These findings underscore MALDI-TOF MS’s potential as a pivotal tool for advancing research into mushroom-derived polysaccharides, with opportunities to enhance their utilization in health-related applications.

### 4.5. MS-Based Analysis Summary

The aim of this section is to streamline the detailed review of mass spectrometry (MS)-based techniques by presenting a concise comparison of their analytical capabilities. This approach is intended to provide readers with a clear and accessible framework for differentiating these techniques based on their suitability for various types of biomass conversion processes. By categorizing and summarizing the MS methods discussed—such as GC-MS, LC-MS, ICP-MS, and MALDI-MS—along with their specific applications to pyrolysis, liquefaction, gasification, and other conversion pathways, this section highlights the strengths and limitations of each method. The goal is to facilitate a quick understanding of which analytical tools are most appropriate for characterizing specific biomass-derived products or reaction intermediates, considering factors such as molecular size, polarity, resolution, sensitivity, and target compounds. Through this synthesis, the section aims to serve as a practical guide for researchers selecting MS techniques for their biomass analysis needs, see Table 1.

## 5. Concluding Remarks

MS-based methods have proven to be indispensable analytical tools across various research domains, including academia and industry. The comprehensive data presented in this review emphasize the transformative potential of advanced mass spectrometry (MS) techniques in the characterization of biomass-derived materials. By leveraging methods such as GC-MS, LC-MS, ICP-MS and MALDI-MS, our study has provided deep insights into the intricate molecular frameworks of materials produced through various biomass conversion processes like pyrolysis and solvent liquefaction. These insights are crucial for the optimization of these processes, contributing significantly to the advancement of sustainable material and energy production.

The detailed reports in this review have profound implications for the industry. By elucidating the composition and impurities in biomass conversion products, MS techniques enable the refinement of production processes, enhance the quality of the outputs, and increase the economic viability of bio-based products. This study highlights key innovations in MS technology, including enhanced sensitivity, specificity, and the ability to analyze complex mixtures, which collectively push the boundaries of what can be achieved in biomass research. In the future, research should focus on expanding the application of MS techniques to emerging areas such as the conversion of non-traditional biomass sources and the development of new bio-based materials with novel properties. There is also a need to refine these MS methods to handle increasingly complex mixtures more efficiently and at a lower cost, which could further increase accessibility to high-quality MS analysis for broader industrial applications.

The contributions of this review extend beyond the technical advances in MS; they encompass a strategic framework for integrating MS into the biomass conversion process lifecycle, from feedstock characterization to end-product validation. The broader relevance of this work lies in its potential to facilitate a shift towards more sustainable industrial practices and to inform regulatory standards and policies on bio-based products. By continuing to build on the foundation this work provides, the field can move towards fully realizing the potential of biomass as a cornerstone of sustainable development.

## Figures and Tables

**Table 1:** Suitable MS-based analysis based on the biomass conversion process.

Conversion Process	Process Characteristics	MS-Based Analysis of the Products	Applications/Challenges
Pyrolysis	A thermochemical process converting biomass into gas mixtures, liquids, and char in the absence or near absence of oxygen [115,116].	GC-MS: Analyzes volatile and semi-volatile compounds, including hydrocarbons, aldehydes, ketones, phenols, and furans [20,21]. ICP-MS: Detects and quantifies trace elements like heavy metals and inorganic impurities in biomass and its derivatives [40,41,42]. Retention times and mass spectra are utilized for the identification of chemical structures.	Biomass-derived biochar; [145] a carbon-rich by-product of pyrolysis, serves as a precursor for biomass activated carbons. Applications include the following areas: - Adsorbents for the removal of environmental pollutants in water [46], air [47] and soil [48]. - Capacitors [49] and supercapacitors [50] for energy storage. - Biologically active materials that exhibit antibacterial, antioxidant [51], and pharmacological properties [52]. - Catalyst supports for electrochemical purposes [53] and biofuel production [54]. - Research highlights impurity analysis’s importance in optimizing applications [72,80,106,116,126]. Catalysts are commonly employed in biomass pyrolysis to reduce the activation energy of reactions and modify the distribution of product components [146]. The pyrolysis process is significantly influenced by parameters like temperature, heating rate, pressure, and residence time, along with the quality and composition of the biomass feedstock, which in turn affect the yield distribution and physicochemical attributes of the final products [147].
Liquefaction	Biomass conversion in a liquid medium, typically under pressure.	LC-MS: Ideal for non-volatile, thermally labile, and structurally complex compounds, including bio-oils, lignin-derived oligomers, and bioactive compounds [34,35,43,128]. MALDI-MS: Useful for analyzing large biomolecules, such as lignin and its derivatives, in biomass-derived products [43,44,45].	Bio-oils, solvent liquefaction products: Impurity analysis helps to improve applications and understand product composition [132,133]. The generation of low-quality bio-crude is characterized by high heteroatom content (e.g., O, N, S), the occurrence of heavy metals, and the intricate management of solid and aqueous by-products. These factors impede process efficiency, sustainability, and scalability, primarily due to diminished heating value and economic viability [148].
Gasification	A thermochemical process for converting biomass into a gaseous product [36].	GC-MS: Analyzes volatile and semi-volatile compounds produced during pyrolysis and gasification [20,21]. ICP-MS: Studies the release patterns of specific elements under varying conditions, providing insights into ash-related challenges and environmental impacts [39]. ICP-MS + TGA: Monitors heavy metal release during gasification [39].	Activated carbons or bio-oils: Impurity analysis is critical for optimizing processes and applications [78]. A minor alteration in process parameters may influence the overall efficacy of the system and the quality of the final product [149]. These parameters include temperature [150], pressure [150], gasifying media [151], air fuel ratio and equivalence ratio [152], residence time [153].
Combustion	Burning biomass materials in the presence of oxygen to produce biomass byproducts. It converts chemicals into thermal energy, which can be applied in different areas.	ICP-MS: Detects and quantifies trace elements, addressing contamination issues and improving process outcomes [40,41,145].	In different studies, [39] ICP-MS is used to analyze behaviors and monitor profiles of certain metals during biomass combustion. Elevated moisture levels, diminished heating values, and the presence of trace elements such as chlorine, sulfur, and alkali metals, leading to fouling, corrosion, ash-related complications, and bed sintering in fluidized beds [154].
